# Alleviating psychosocial distress in drug-resistant tuberculosis: a pre-post study on telephone-delivered motivational interviewing

**DOI:** 10.3389/fpubh.2026.1817457

**Published:** 2026-05-08

**Authors:** Fanghui Xie, Enchun Xie, Xiaoyi Yang, Bin Wan, Rong Yao

**Affiliations:** 1 Department of Tuberculosis, Public Health Clinical Center of Chengdu, Chengdu, Sichuan, China; 2 Department of Nursing, Public Health Clinical Center of Chengdu, Chengdu, Sichuan, China

**Keywords:** anxiety, depression, drug-resistant tuberculosis, loneliness, medication adherence, motivational interviewing, sleep quality, telephone intervention

## Abstract

**Objective:**

This pilot feasibility study aimed to evaluate the efficacy of a telephone-delivered motivational interviewing (MI) intervention in alleviating loneliness, depressive symptoms, and anxiety among patients with drug-resistant tuberculosis (DR-TB).

**Methods:**

Employing a single-group pre-post design, 74 DR-TB patients were recruited from the tuberculosis wards of Chengdu Public Health Clinical Medical Center between January and December 2021. Participants underwent a 12-week MI-based telephone intervention, with assessments at baseline (Week 0) and post-intervention (Week 16). Primary outcomes included changes in psychological measures using validated instruments: the University of California, Los Angeles Loneliness Scale (UCLA-LS), Self-Rating Depression Scale (SDS), and Hamilton Anxiety Rating Scale (HAMA). Secondary outcomes encompassed sleep quality, assessed by the Pittsburgh Sleep Quality Index (PSQI), and medication adherence, defined as a binary outcome based on pill count.

**Results:**

The intervention significantly reduced loneliness (UCLA-LS: 47.23 ± 10.14 → 40.23 ± 12.76, *p* < 0.001; 14.8% decrease), anxiety (HAMA: 14.73 ± 8.85 → 12.06 ± 7.34, *p* = 0.017; 18.1% reduction), and depressive symptoms (SDS: 50.47 ± 9.33 → 34.85 ± 9.06, *p* < 0.001; 30.9% decline). Sleep quality improved (PSQI: 7.75 ± 0.68 → 4.20 ± 1.44, *p* < 0.001; 45.8% decline), while medication adherence increased from 89.2 to 95.4%, this change was not statistically significant (*p* > 0.05, McNemar’s test).

**Conclusion:**

This pilot pre-post study provides preliminary evidence that telephone-delivered MI is associated with improved psychological outcomes in DR-TB patients. The findings support its promise as an accessible intervention, but the exploratory design necessitates further investigation through controlled trials to confirm efficacy and establish causal inference.

## 1 Introduction

### 1.1 Background and global burden of DR-TB

Managing drug-resistant tuberculosis (DR-TB) continues to pose substantial global health burdens. Despite progress through DOTS implementation, WHO 2024 data indicate only 44% of Multidrug-Resistant/Rifampicin-Resistant Tuberculosis (MDR/RR-TB) patients worldwide receive appropriate therapy (1). This gap is particularly pronounced in high-burden countries such as China, which accounted for 7.3% of global cases (29,000 cases) in 2023 (1). Although new treatment regimens have improved the global treatment success rate to 68% (1), DR-TB management continues to be complicated by multifaceted challenges, including high treatment non-adherence rates (46.52%) (2), frequent adverse drug reactions (71.3% of patients experience at least one adverse event) (3), and the prevalence of severe psychosocial comorbidities, which affect over 60% of patients (4).

### 1.2 Psychosocial burden of DR-TB

DR-TB patients often face significant psychosocial distress, including loneliness, anxiety, and depression, which are exacerbated by the social isolation and stigma associated with the disease (5). Studies have shown that 56.2% of DR-TB patients reported experiencing a sense of loneliness (6), with anxiety and depression prevalence rates ranging from 6.8 to 54.6% and 7.8 to 55.9%, respectively (7–9). In recent years, a study in Southern China revealed that 57.5% of MDR/RR-TB patients experienced anxiety, 65.8% experienced depression, and 33.3% suffered from both conditions simultaneously (10). Similarly, in Indonesia, 86.3% of newly diagnosed MDR-TB patients reported anxiety, with 6.9% experiencing extreme anxiety (11). These psychosocial challenges impair patients’ quality of life and contribute to treatment non-adherence and loss to follow-up, further complicating DR-TB management (12, 13). Recent qualitative studies have further corroborated that patients with DR-TB experience multifaceted psychosocial maladjustment stemming from challenges in social interactions, medication-related issues, economic constraints, societal pressures, and family dynamics, which collectively compromise medication adherence and impede treatment progress (14, 15).

### 1.3 Current gaps in psychosocial support for DR-TB patients

Although robust evidence confirms that psychosocial stressors adversely affect DR-TB treatment outcomes, clinical guidelines still prioritize screening protocols, diagnostic pathways, and therapeutic regimens. Systematic incorporation of psychological support to meet patients’ holistic health requirements remains limited. Recognizing this gap, global health authorities advocate for integrated psychosocial care: The International Union Against Tuberculosis and Lung Disease (The Union) urges inclusion of psychosocial support (PSS) in TB management programs (16), while WHO’s 2022 operational handbook specifically endorses psychological counseling (17). Nevertheless, empirically validated psychosocial interventions—particularly in resource-constrained settings (Low- and Middle-Income Countries, LMICs)—continue to be underdeveloped.

### 1.4 Motivational interviewing as a potential intervention

MI is a patient-centered communication approach that fosters collaboration between healthcare providers and patients, aiming to enhance intrinsic motivation and promote behavioral change (18). Rooted in Carl Rogers’ humanistic theory, MI integrates social and cognitive psychology to assess a patient’s readiness to change and resolve ambivalence toward behavioral modification (19). It is defined by four successive processes: (i) engagement, establishing mutual trust and respect; (ii) focusing, identifying behavioral objectives to guide subsequent sessions; (iii) evocation, exploring the patient’s motivation and readiness for change; and (iv) planning, collaboratively developing an action plan when the patient is prepared to commit to change. Each session progressively cultivates empathy and collaboration, applying cognitive strategies such as open-ended questions, affirmations, and reflective listening (20).

A study involving 275 women living with HIV demonstrated that four MI sessions (conducted at 2, 6, 10, and 24 weeks post-enrollment) combined with text message reminders effectively supported breastfeeding and improved infant health outcomes (21). MI has been effectively applied in chronic disease management, including heart failure and cancer, where it improves self-care, physical activity, and psychological well-being (22, 23). Recent literature has further expanded MI’s application to the six pillars of lifestyle medicine—healthy diet, mental health, healthy interpersonal relationships, physical activity, reduction of harmful substances, and sleep—demonstrating its versatility in promoting positive behavioral change (24). In the context of tuberculosis (TB), MI has demonstrated potential in enhancing medication adherence and treatment success by addressing treatment barriers and fostering patient empowerment (25).

Building on this foundation, the integration of MI with remote technologies has opened new possibilities for psychosocial support, particularly in resource-limited settings. Recent studies have demonstrated the feasibility of synchronous video-counseling for TB patients and asynchronous mHealth interventions for HIV, underscoring technology’s potential to deliver structured psychological support (26, 27). Telemedicine transcends geographic and temporal barriers, and hybrid models with professional supervision show enhanced acceptability among TB patients in resource-constrained settings (28). Regarding implementation, MI interventions typically require trained healthcare workers who have completed supervised practice and fidelity monitoring (29). In this study, the interventionist completed a 12-week specialized training program in respiratory infectious disease nursing and underwent rigorous MI training including simulation exercises (detailed in Section 2.3.1), with ongoing fidelity review by quality control personnel. However, the application of MI in DR-TB management, particularly through telephone-based interventions, remains underexplored.

In China, where DR-TB patients primarily receive home-based treatment, telephone follow-up is common but focuses on medication adherence and adverse drug reactions, with limited attention to psychological needs. This study aims to bridge this gap by evaluating a telephone-delivered MI intervention for alleviating loneliness, depression, and anxiety among DR-TB patients. By operationalizing WHO’s psychosocial care guidelines in home-treatment settings, this study seeks to establish evidence for technology-mediated MI in resource-constrained environments and inform the integration of psychological support into routine TB care protocols.

## 2 Methods

### 2.1 Study design

This study employed a single-group pre-post pilot design to evaluate the effects of a telephone-delivered motivational interviewing intervention on psychosocial distress in patients with drug-resistant tuberculosis. The pre-post design was intentionally selected for this pilot feasibility study for several reasons: (1) to evaluate the feasibility and acceptability of the telephone-delivered MI intervention in a real-world clinical setting prior to conducting a larger, resource-intensive randomized controlled trial; (2) to estimate effect sizes for key psychosocial outcomes to inform sample size calculations for future definitive trials; (3) to identify potential implementation challenges and refine the intervention protocol; and (4) to assess recruitment capacity and retention rates in the target population. This self-controlled before-and-after trial evaluated the effects of MI-based telephone follow-up on loneliness, depression, and anxiety among DR-TB patients. The intervention involved integrating MI-based telephone follow-up services into routine patient management protocols, divided into pre-intervention, intervention, and post-intervention phases. This study was reported following the Strengthening the Reporting of Observational Studies in Epidemiology (STROBE) guidelines (see Supplementary Table S1).

### 2.2 Participants

From January to December 2021, inpatients with drug-resistant tuberculosis were recruited from Chengdu Public Health Clinical Medical Center, which is situated in Sichuan Province in western China, with a total of 80 patients enrolled.

Clinical pathway and enrolment timeline: All patients were first admitted to the tuberculosis wards of our hospital, where their all-oral chemotherapy regimen was determined and the first doses of anti-tuberculosis medications were administered under supervision. Following discharge, patients were referred to our Drug-Resistant Tuberculosis Follow-up Center for routine outpatient management. Enrolment into this study occurred after discharge, at the time patients entered the follow-up center. Therefore, the baseline assessment (Week 0) and the first telephone MI session (Week 2) took place while patients were already in the community-based treatment phase. This explains the variation in treatment duration at enrolment (see Section 3.1), as patients were referred to our follow-up center at different points in their treatment journey—some shortly after initial diagnosis, others after transfer from other facilities or later stages of their regimen.

Whether patients were eligible for the study was judged according to set inclusion and exclusion criteria. The inclusion criteria covered having confirmed pulmonary tuberculosis resistant to at least rifampicin, being 18–65 years old, receiving an all-oral chemotherapy regimen, and providing written informed consent (see Supplementary Table S2). Participants were treated with standardized, longer-term all-oral regimens for MDR/RR-TB, in accordance with contemporaneous WHO guidelines. These regimens typically had a duration of 18–24 months and comprised a combination of drugs such as Bedaquiline, Levofloxacin, Linezolid, Cycloserine, and Clofazimine. Exclusion criteria included psychiatric disorders impairing communication, vital organ insufficiency, current psychotherapy, poor compliance, and incomplete clinical data.

### 2.3 Intervention

The interventionist, trained in MI, conducted four telephone follow-up sessions over 12 weeks. Each session lasted 30–40 min, focusing on engaging, focusing, evoking, and planning phases to address patients’ psychological needs and promote behavioral change. To enhance replicability, detailed examples of common patient concerns, counselor questions, and specific MI strategies are described below.

#### 2.3.1 Phase 1: pre-intervention preparation

The interventionist underwent rigorous training in MI techniques prior to the study. This training included a comprehensive review of the Motivational Interviewing Manual (30), video demonstrations, and an in-depth analysis of relevant literature. The interventionist, who had 5 years of experience in telephone follow-up for DR-TB patients, also completed a 12-week specialized training program in respiratory infectious disease nursing and held relevant certifications. To ensure proficiency, the interventionist engaged in simulation exercises, which were designed to replicate real-world scenarios and enhance the application of MI principles.

#### 2.3.2 Phase 2: intervention implementation

The intervention was conducted over a 12-week period, during which four telephone follow-up sessions were scheduled at Weeks 2, 4, 8, and 12. Each session lasted between 30 to 40 min and was meticulously designed to address the psychological needs of DR-TB patients while promoting behavioral change. The interventionist followed a structured protocol to ensure consistency and fidelity across all sessions.

Engaging Phase (Approximately 5 min):

The interventionist established a quiet and confidential communication environment to foster trust and openness. Through open-ended questions and active listening, the interventionist gathered detailed information about the patient’s psychological state, treatment experiences, and concerns. This phase also included providing basic education about DR-TB to enhance the patient’s understanding of their condition and treatment.

*Common* questions included: “What has been the hardest part of your treatment so far?” and “How have you been feeling emotionally since starting the medication?”

*Frequent* patient concerns included fear of transmitting the disease to family members, discomfort from medication side effects (e.g., nausea, fatigue), and feelings of isolation due to prolonged home-based treatment.

Focusing Phase (Approximately 10 min):

The conversation was gradually shifted toward positive psychology, encouraging patients to share their psychological changes and concerns related to their illness. The interventionist provided psychological comfort and emphasized the importance of maintaining a positive mental state for TB treatment outcomes and overall health. Collaborative problem-solving was employed to address specific issues, maximizing emotional support and reinforcing the patient’s ability to cope with their condition.

*Example* strategies: The interventionist would ask, “What do you think would help you feel more in control of your emotions?” and reflect, “It sounds like you’re worried about how your family sees you right now—many patients share that concern.”

Evoking Phase (Approximately 10 min):

The interventionist attentively listened to the patient’s narrative, identifying internal conflicts and ambivalence. Change talk was elicited and reinforced to motivate the patient to improve their psychological state and enhance their confidence in facing the illness. This phase aimed to strengthen the patient’s intrinsic motivation for behavioral change by highlighting their own reasons for wanting to improve their mental health.

*Specific* MI techniques included: exploring discrepancies between personal goals and current behaviors, affirming patient strengths, and using scaling questions such as, “On a scale from 0 to 10, how important is it for you to stay on track with your treatment, and why are you at that number rather than a lower one?”

Planning Phase (Approximately 10 min):

As the patient increasingly discussed the possibility of change, the interventionist assessed their readiness to develop an action plan. Potential psychological barriers were identified, and problem-solving strategies were explored, such as accessible relaxation techniques and rewards for achieving goals. Patients were invited to describe their action plans, and joint goals for improving mental health were set. An electronic version of the Psychological Relaxation Education Manual was provided to each patient via the internet to support their ongoing efforts.

*Example* collaborative goal: One patient who expressed loneliness agreed to call a close friend twice a week and practice a 5-min breathing exercise daily, which they later reported as helpful.

Illustrative Case Example:

A 34-year-old male patient, 3 months into treatment, expressed frustration about nausea and fear that his neighbors had learned of his diagnosis. During the Engaging phase, he shared, “I feel like everyone is avoiding me.” In the Focusing phase, the interventionist reflected, “It sounds like the fear of being judged is weighing on you.” During Evoking, the patient stated, “I want to finish treatment so I can be healthy and protect my family.” This change talk was reinforced, and in the Planning phase, the patient developed a plan to take medication after dinner to reduce nausea and to speak with one trusted neighbor about his situation. At the Week 12 session, he reported reduced anxiety and improved adherence.

#### 2.3.3 Phase 3: post-intervention assessment

At Week 16, treatment fidelity was assessed, and data on patients’ loneliness, depression, anxiety, sleep quality, and medication adherence were collected. The interventionist reviewed the follow-up records to evaluate the quality of the intervention and optimize the process and content based on the assessment results. This phase ensured that the intervention’s effectiveness was maintained and that any necessary adjustments were made to enhance patient outcomes.

Quality control personnel regularly reviewed the follow-up records to assess the intervention’s fidelity and effectiveness. Any deviations from the protocol were identified and addressed promptly to ensure the integrity of the intervention. The interventionist received ongoing feedback and support to maintain high standards of MI application throughout the study.

### 2.4 Measures

The main outcomes assessed were variations in loneliness, measured using the UCLA loneliness scale (UCLA-LS); anxiety, evaluated via the Hamilton anxiety scale (HAMA); and depressive symptoms, gaged with the self-rating depression scale (SDS). The secondary outcomes encompassed sleep quality, as determined by the Pittsburgh sleep quality index (PSQI), and medication adherence, assessed through the objective, binary outcome based on pill count. All questionnaires are presented in Supplementary Table S2.

#### 2.4.1 Loneliness

This study adopted the third edition of the UCLA Loneliness Scale, which was created by Russell and colleagues (31). This unidimensional scale consists of 20 items, among which items 1, 5, 6, 9, 10, 15, 16, 19, and 20 are reverse-scored. It uses a 4-point Likert scale, with each item scored from 0 to 3, leading to a total score ranging from 20 to 80. A higher total score signifies a stronger sense of loneliness. The Cronbach’s alpha coefficient of this scale was 0.936, indicating that it has good reliability and validity.

#### 2.4.2 Anxiety

The HAMA, initially developed by Hamilton (32), was adapted into a Chinese version by Zhang Mingyuan (33). It comprises 14 items, each scored on a 0–4 scale that corresponds to the symptom levels of “asymptomatic,” “mild,” “moderate,” “severe,” and “extremely severe.” The total score ranges from 0 to 56, with higher scores reflecting more severe anxiety. The Chinese version of HAMA showed excellent reliability, with a Cronbach’s alpha coefficient of 0.930.

#### 2.4.3 Depression

The SDS, developed by Zung in 1965 and adapted for the Chinese population by Shu Liang, is used to evaluate a patient’s depressive state over the past week (34). It contains 20 items, 10 of which are reverse-scored, and uses a 4-point Likert scale. The sum of the item scores is the raw total score, and the standard score is calculated by multiplying the raw total score by 1.25 and taking the integer part. The cut-off values for depression are as follows: a score below 53 indicates no depression; 53–62 indicates mild depression; 63–72 indicates moderate depression; and a score above 72 indicates severe depression.

#### 2.4.4 Sleep quality

The PSQI was employed to assess sleep quality. First developed by Buysse and colleagues in 1989, this scale was later adapted for the Chinese population by Liu Xianchen and others (35). It is designed to evaluate participants’ sleep quality over the preceding month and consists of 18 self-rated items across seven dimensions: subjective sleep quality, sleep latency, sleep duration, sleep efficiency, sleep disturbances, use of hypnotic drugs, and daytime dysfunction. The total score ranges from 0 to 21, with higher scores signifying worse sleep quality. For the Chinese version of the PSQI, the Cronbach’s *α* coefficients for each dimension fall between 0.842 and 0.852.

#### 2.4.5 Medication adherence

Medication adherence was assessed using an objective, binary outcome based on pill count. Patients were considered “regular adherers” if they had taken 90% or more of their prescribed pills during the study period (36). Adherence was calculated using the formula: [(Number of pills prescribed - Number of pills remaining) / Number of pills prescribed] × 100%. Patients with an adherence rate of ≥90% were categorized as regular adherers; those with a rate of <90% were categorized as non-adherers.

### 2.5 Data collection

Data collection was conducted at two time points: baseline (Week 0) and endline (Week 16). Baseline assessments were completed prior to the first telephone MI session, capturing pre-intervention status without any influence from the intervention. Endline assessments were conducted 4 weeks after the final intervention session (Week 12) to evaluate sustained effects rather than immediate post-session reactivity. No intermediate assessments were performed during the 12-week intervention period, a decision made to minimize participant burden given the demanding nature of DR-TB treatment and to maintain the feasibility focus of this exploratory study.

At baseline, a comprehensive evaluation of patients’ characteristics was performed, covering demographic information and clinical indicators, including age, gender, education level, marital status, economic condition, duration of DR-TB treatment, presence of concurrent chronic diseases, body mass index (BMI), and other related social demographic and clinical features. In addition, initial assessments were made on loneliness, depression, anxiety, sleep quality, and medication adherence. This timeline was applied uniformly to all participants regardless of their treatment stage at enrollment, ensuring consistency in the evaluation period.

To ensure data accuracy and integrity, all collected information was independently verified by two research team members. Once confirmed to be error-free, the data were systematically entered into Excel for subsequent analysis.

### 2.6 Statistical analysis

SPSS 26.0 software was utilized for data analysis (see Supplementary data S1, S2). Measurement data were presented as mean ± standard deviation (^−^x ± S), while qualitative data were expressed by the number of cases and percentage (%). Paired-sample t-tests were employed to compare the scores before and after the intervention. For categorical data, differences between pre- and post-intervention assessments were compared using the Chi-square test for independent samples, while the McNemar test was applied for paired comparisons (i.e., medication adherence). Effect sizes (Cohen’s d) were computed, and a *p*-value less than 0.05 was regarded as statistically significant.

Sample Size Justification: As this was a pilot feasibility study, a formal *a priori* sample size calculation was not performed. The sample size was determined by the number of eligible DR-TB patients who were admitted to the Chengdu Public Health Clinical Medical Center and consented to participate during the predefined 12-month recruitment window (January to December 2021). This approach is consistent with the primary goal of pilot studies to assess feasibility and estimate effect sizes for future definitive trials. We acknowledge that this sample size may have limited statistical power to detect smaller effects, and findings should be interpreted as preliminary.

No adjustment for multiple comparisons was applied to the alpha level (*α* = 0.05) for the primary outcome analyses. This decision was based on the exploratory, hypothesis-generating nature of this pilot study and the desire to avoid increasing Type II error rates for the pre-specified, inter-related psychological outcomes. However, the unadjusted *p*-values are reported transparently and should be interpreted with this in mind. As a sensitivity analysis, a Bonferroni correction was also considered (see Results).

### 2.7 Consideration of bias and confounding

As a single-arm pre-post study, several potential sources of bias and confounding were considered. Selection bias is a key consideration, as participants were recruited from a single clinical center. The notably high educational attainment of our sample (54.05% with a college degree or higher) may limit the generalizability of the findings to DR-TB populations with lower education levels or different socioeconomic backgrounds. Information bias, such as social desirability bias in self-reported psychological and adherence measures, was possible. Furthermore, the absence of a control group means that the effects observed could be influenced by unmeasured confounders or external factors coinciding with the intervention period.

To mitigate these concerns, several steps were taken. We employed standardized and validated instruments to ensure the consistency and reliability of our measurements. All data were double-checked by two independent research team members to minimize entry errors. The primary analysis focused on within-subject changes to control for time-invariant patient characteristics. It is important to interpret the findings in the context of this being a feasibility study, which aims to generate preliminary evidence for future controlled trials that can better address these methodological limitations.

### 2.8 Ethical considerations

This study was conducted in accordance with the principles of the Declaration of Helsinki and was approved by the Ethics Committee of The Public Health Clinical Center of Chengdu (Approval No.: PJ-K2020-56-01). Informed consent was obtained from all participants before data collection.

The informed consent process was conducted as follows: after patients were discharged from the tuberculosis wards and entered the Drug-Resistant Tuberculosis Follow-up Center, trained research staff explained the study purpose, procedures, potential risks and benefits, voluntary participation, and the right to withdraw at any time without affecting their clinical care. Written informed consent was obtained from each participant prior to any study-related procedures. To ensure confidentiality, all data were anonymized using unique study identifiers, and only the research team had access to identifiable information.

## 3 Results

### 3.1 Participant characteristics

A total of 74 patients with drug-resistant tuberculosis completed the study (retention rate: 92.50%; 6 patients were lost to follow-up: 3 withdrew consent due to lack of interest, and 3 discontinued treatment due to financial constraints). The baseline demographic and clinical characteristics of the participants are summarized in Table 1.

**Table 1:** Baseline demographic and clinical characteristics of participants (*N* = 74).

Characteristic	Category	n (%)
Age (years)	18–30	38 (51.4)
	31–45	22 (29.7)
	46–65	14 (18.9)
	Mean ± SD	31.85 ± 10.89
Gender	Male	42 (56.8)
	Female	32 (43.2)
Education level	Elementary school or lower	8 (10.8)
	Junior high school	18 (24.3)
	High school or vocational school	8 (10.8)
	College or bachelor’s degree	39 (52.7)
	Graduate degree or higher	1 (1.4)
Marital status	Unmarried	35 (47.3)
	Married	33 (44.6)
	Divorced or widowed	6 (8.1)
Monthly income (yuan)	<1,500	47 (63.5)
	1,500–3,000	8 (10.8)
	3,000–4,500	8 (10.8)
	>4,500	11 (14.9)
Treatment duration at enrollment	<6 months	53 (71.6)
	6 months-1 year	4 (5.4)
	>1 year	17 (23.0)
BMI (kg/m^2^)	Underweight (<18.5)	35 (47.3)
	Normal (18.5–24.9)	33 (44.6)
	Overweight (≥25.0)	6 (8.1)
	Mean ± SD	19.98 ± 2.84

The cohort comprised 42 males (56.80%) and 32 females (43.20%), with a mean age of 31.85 years (±10.89). Regarding treatment duration at enrollment, 53 patients (71.6%) had been receiving DR-TB treatment for less than 6 months, 4 patients (5.4%) for 6 months to 1 year, and 17 patients (23.0%) for more than 1 year. None of the participants had comorbid chronic diseases. This finding may be explained by the following factors: (1) the mean age of the sample was 31.85 years, with the majority of participants being relatively young adults; (2) patients with significant chronic comorbidities such as diabetes mellitus, chronic kidney disease, or HIV infection are often managed in specialized clinics or referral centers separate from our tuberculosis ward; and (3) the study’s exclusion criteria included vital organ insufficiency, which may have preferentially excluded patients with significant chronic conditions. Additionally, while patients with stable chronic conditions were not actively excluded, their representation in this cohort was limited.

Regarding treatment-related adverse effects, no severe adverse events requiring hospitalization or regimen modification were reported during the study period. Common mild to moderate manageable side effects were monitored as part of routine care.

### 3.2 Intervention effects

The effects of the intervention on psychological status, sleep quality, and medication adherence are summarized in Tables 2–4.

**Table 2:** Changes in psychological and health-related indicators before and after intervention.

Indicator	Pre-intervention	Post-intervention	Change (%)	Direction
Loneliness (UCLA-LS)	47.23	40.23	−14.8%	Decrease
Anxiety (HAMA)	14.73	12.06	−18.1%	Decrease
Depression (SDS)	50.47	34.85	−30.9%	Decrease
sleep quality (PSQI)	7.75	4.20	−45.8%	Decrease

**Table 3:** Statistical analysis of intervention effects.

Indicator	Baseline (Mean ± SD)	Post-intervention (Mean ± SD)	Mean difference (95% CI)	*p*-value	Cohen’s *d*	Effect size
Loneliness (UCLA-LS)	47.23 ± 10.14	40.23 ± 12.76	+7.00	<0.001	0.60	Moderate
Anxiety (HAMA)	14.73 ± 8.85	12.06 ± 7.34	+2.67	0.017	0.32	Small
Depression (SDS)	50.47 ± 9.33	34.85 ± 9.06	+15.62	<0.001	1.65	Large
Sleep quality (PSQI)	7.75 ± 0.68	4.20 ± 1.44	+3.55	<0.001	1.24	Large

**Table 4:** Comparison of patients’ medication adherence (assessed by pill count) before and after the intervention (*n* = 74).

Time point	Regular adherers	Non-adherers	χ^2^	*p*
Pre-intervention	66 (89.2%)	8 (10.8%)	3.2	>0.05
Post-intervention	71 (95.4%)	3 (4.6%)		

McNemar’s test was used.

The pre-post intervention was associated with significant improvements in scores for loneliness, anxiety, depression, and sleep quality in unadjusted analyses (all *p* < 0.05). The largest effects were observed for depression (Cohen’s *d* = 1.65) and sleep quality (Cohen’s *d* = 1.24), both indicating large effect sizes. When applying a more conservative Bonferroni correction for multiple comparisons (significance threshold set at *p* < 0.01), the improvements in loneliness, depression, and sleep quality remained statistically significant, while the reduction in anxiety did not.

Clinical interpretation of the observed changes:

Using established cut-off scores, the improvements translated into meaningful clinical shifts. For depression, the mean SDS score decreased from 50.47 (±9.33), which falls within the mild depression range (standardized scores 53–62), to 34.85 (±9.06), which is below the depression threshold (<53), indicating that the average patient moved from a clinically significant depressive state to a non-clinical range. For anxiety, the mean HAMA score decreased from 14.73 (±8.85), corresponding to mild to moderate anxiety (scores ≥14 indicate clinical significance), to 12.06 (±7.34), falling below the clinical cut-off. For sleep quality, the mean PSQI score improved from 7.75 (±0.68), reflecting clinically significant poor sleep (>5), to 4.20 (±1.44), which is within the good sleep quality range (≤5). These shifts suggest that the intervention not only produced statistically significant improvements but also helped the average patient achieve symptom levels no longer meeting clinical thresholds for depression, anxiety, and sleep disturbance.

Following the intervention, the rate of regular medication adherence among patients increased from 89.2% (66/74) at baseline to 95.4% (71/74). However, McNemar’s test indicated no statistically significant change in medication adherence (*p* > 0.05), likely due to a ceiling effect given the high baseline adherence.

## 4 Discussion

This pre-post study provides preliminary evidence that a telephone-delivered MI intervention was associated with significant improvements in loneliness, anxiety, depressive symptoms, and sleep quality among patients with DR-TB. The primary null finding—that no statistically significant change in objectively measured medication adherence was observed—offers a critical nuance for interpreting the intervention’s impact and underscores the value of rigorous measurement in behavioral research.

### 4.1 Interpretation of key findings

The substantial improvements in psychosocial outcomes align well with the core mechanisms of MI. The structured telephone sessions, employing open-ended questions, active listening, and collaborative planning, likely created a therapeutic environment where patients felt empowered to articulate emotional distress and identify their own motivations for psychological adjustment. This patient-centered approach minimizes resistance and fosters intrinsic motivation, which is directly applicable to managing feelings of loneliness, anxiety, and depression (37, 38).

Importantly, these statistically significant improvements also carried meaningful clinical implications. For depression, the mean SDS score fell from the mild depression range (standardized scores 53–62) at baseline to below the diagnostic threshold (<53) post-intervention, indicating that the average patient moved from a clinically significant depressive state to a non-clinical range. Similarly, the mean HAMA score decreased from a level consistent with mild to moderate anxiety (≥14) to below the clinical cut-off, reflecting a reduction in clinically significant anxiety symptoms. These shifts suggest that the intervention helped patients achieve symptom levels no longer meeting clinical thresholds, which is likely to translate into tangible benefits such as improved daily functioning, reduced emotional burden, and enhanced capacity to engage with long-term treatment (12, 13). These findings are consistent with evidence that technology-delivered motivational interviewing can improve depression and anxiety outcomes in patients with chronic conditions (39, 40).

The concurrent, marked improvement in sleep quality further supports the clinical relevance of the intervention. The mean PSQI score improved from clinically significant poor sleep (>5) at baseline to within the good sleep quality range (≤5) post-intervention. Given the well-established bidirectional relationship between mental health and sleep, this improvement is consistent with the observed reductions in depression and anxiety, and may contribute to a positive feedback loop that sustains psychological well-being (41, 42). The effect size for sleep quality (Cohen’s *d* = 1.24) exceeds those reported in many non-pharmacological sleep interventions, suggesting that addressing underlying psychological distress may be a particularly effective pathway for improving sleep in this population.

Regarding loneliness, Adejumo and colleagues reported that 56.2% of DR-TB patients in Nigeria experienced loneliness, with stigma and social isolation being key contributing factors (6). Our intervention achieved a 14.8% reduction in UCLA-LS scores, demonstrating that telephone-based MI can effectively address the social isolation inherent to DR-TB treatment. This reduction compares favorably with psychosocial interventions for loneliness in other chronic disease populations. Anxiety and depressive symptoms can significantly impair an individual’s capacity to engage in meaningful activities, particularly those involving social interaction, which are crucial for deriving a sense of purpose and connection (43). This reduced engagement may stem from a dual mechanism: these psychological states can diminish motivation for participation and disrupt value-driven behavior, steering individuals away from activities aligned with their core values or hindering full immersion in chosen pursuits (44). Consequently, as participants’ anxiety and depressive symptoms improved in this study, their sense of loneliness decreased accordingly.

### 4.2 Contextualizing the null finding on medication adherence

The lack of a significant change in medication adherence, as measured by the objective pill count, is a pivotal finding. This is best explained by a pronounced ceiling effect. Participants exhibited exceptionally high baseline adherence (89.2%), leaving minimal room for statistically meaningful improvement. This high baseline is contextually understandable: over 50% of participants held a university degree or higher, a demographic segment typically associated with elevated health literacy and more optimal health behaviors (45). Consequently, while MI has been shown to improve adherence in populations with suboptimal baseline behavior, its effect in this already highly adherent cohort was inherently attenuated. Notably, the post-intervention adherence rate remained above 95%—a clinically desirable outcome—even though the change was not statistically significant.

MI is a patient-centered approach designed to address ambivalence and enhance intrinsic motivation for change, particularly among those not yet ready to modify their behavior (46, 47). In this study, the null finding suggests that for patients with already optimal adherence—likely due to high health literacy—the intervention’s primary value may lie elsewhere. Therefore, this result reframes MI’s success in this context: its principal benefit was in addressing the concomitant psychosocial burden of the disease rather than further boosting an already optimal behavior.

This finding also highlights a crucial methodological consideration. The use of an objective adherence measure (pill count) prevented the overestimation of interventional effects that can occur with subjective self-reports. Future research should target patients with demonstrated adherence challenges, where the potential for MI to effect meaningful change is greatest.

### 4.3 Theoretical framework and implications for practice

Our intervention can be conceptualized within the Capacity-Motivation-Opportunity (CMO) framework for patient-centered care (48, 49). In this cohort, the telephone-delivered MI sessions primarily acted on the motivation component, enhancing intrinsic drive and self-efficacy for psychological self-management. Simultaneously, they built patient capacity through education and problem-solving skills (e.g., relaxation techniques, action planning), and leveraged the opportunity provided by accessible telecommunication technology, which overcame barriers of geographic distance and infection control concerns.

The results suggest that for patients with high pre-existing adherence capacity, MI’s primary benefit lies in addressing the concomitant psychosocial burden of disease rather than further boosting an already optimal behavior. From a clinical practice perspective, the intervention demonstrated that a brief, telephone-delivered MI protocol can achieve meaningful psychological improvements—moving the average patient from clinically significant to non-clinical ranges for depression, anxiety, and sleep disturbance. For TB programs, particularly in resource-limited settings, this offers a feasible and scalable strategy to address the holistic needs of DR-TB patients without requiring extensive in-person mental health infrastructure. The telephone modality is particularly advantageous where geographic distance, transportation barriers, or infection control concerns limit access to in-person care (23, 25).

In summary, this reframes the intervention’s success as a holistic support tool that complements—rather than merely drives—pharmacological treatment. These findings lay the groundwork for future research to explore which patients benefit most from this approach, as discussed below.

### 4.4 Future directions and subgroup considerations

Although this feasibility study was not powered for subgroup analyses, identifying which patients derive the greatest benefit from telephone-delivered MI remains a priority for future research. Based on the demographic and clinical characteristics of our cohort, several patient subgroups warrant specific investigation.

Age-related considerations. Younger patients may experience greater loneliness and benefit more from remote psychosocial support, given their higher digital engagement. In contrast, older patients may face unique barriers, such as lower technological literacy or greater social isolation due to mobility limitations (50). Future studies should examine whether intervention effects differ by age group and whether age-specific adaptations (e.g., simplified instructions for older adults) are needed.

Educational level. Our sample possessed a notably high education level (over 50% held a university degree or higher), which may have contributed to the high baseline adherence and responsiveness to MI. Patients with lower educational attainment may have different health literacy needs and thus benefit from adapted communication strategies. Investigating whether the intervention’s effectiveness varies by education level would help tailor implementation strategies for diverse populations.

Gender differences. Previous TB research has shown that women often report higher levels of stigma and psychological distress, potentially influencing their responsiveness to patient-centered counseling (51). Exploring gender differences in intervention response could inform gender-sensitive psychosocial support approaches.

Treatment stage. Patients at different stages of treatment—early versus late in their DR-TB course—may present with distinct psychosocial needs. Those newly diagnosed may grapple with stigma and treatment acceptance, while those in later stages may contend with treatment fatigue and cumulative side effects (14). In our cohort, the heterogeneity in treatment duration at enrollment (71.6% within 6 months, 23.0% beyond 1 year) reflects real-world clinical diversity. Exploring whether intervention effects differ by treatment stage could help optimize the timing of psychosocial support delivery.

Descriptive observations by treatment stage. Although formal subgroup analyses were not performed due to the pilot nature of this study, a descriptive examination of baseline data suggests that patients earlier in their treatment course (<6 months) reported numerically higher levels of depression, anxiety, and loneliness compared to those who had been on treatment for more than 1 year. This observation, while not statistically tested, raises the hypothesis that psychological distress may be more pronounced in the early phase of DR-TB treatment, potentially due to the initial shock of diagnosis, stigma, and treatment adjustment. However, patients in later stages may face other challenges such as treatment fatigue and cumulative side effects. Therefore, while early-stage psychosocial support may be particularly important, ongoing support throughout the treatment journey is still warranted. These descriptive observations should be interpreted as hypothesis-generating and confirmed in future adequately powered studies.

Other potential moderators. Beyond the factors above, baseline symptom severity, social support level, and comorbidity status may also influence intervention response and warrant investigation in future studies.

Future adequately powered randomized controlled trials should prospectively examine these potential moderators using pre-specified subgroup analyses or interaction tests. Such efforts will guide the targeted, patient-centered implementation of MI-based interventions in DR-TB care.

### 4.5 Alternative explanations and potential confounders

While the observed improvements are consistent with the hypothesized benefits of MI, several alternative explanations warrant consideration.

First, natural history and temporal effects could explain some of the changes, as psychological symptoms in chronic illness can fluctuate over time. However, the substantial magnitude of improvement—particularly the shift from clinically significant to non-clinical ranges for depression, anxiety, and sleep quality—argues against simple regression to the mean or natural recovery as the sole explanation.

Second, a Hawthorne effect may be at play. Participants in single-group designs may experience increased attention from healthcare providers, which can enhance psychological well-being regardless of the specific intervention content. The regular telephone contacts (four sessions over 12 weeks) may have provided a sense of being cared for and monitored.

Third, concurrent clinical care—including ongoing DR-TB treatment and routine adverse event follow-up—may have improved physical symptoms, which could secondarily enhance psychological outcomes.

Fourth, the high educational attainment of our sample (over 50% held a university degree or higher) may have contributed to greater health literacy and self-management skills. Such individuals may be more responsive to behavioral interventions and self-report measures or employ effective coping strategies even without MI.

Fifth, placebo and expectation effects cannot be ruled out. Participants enrolled in a research study may hold positive expectations about the intervention, potentially influencing self-reported improvements.

Given the pilot nature of this study, these findings should be interpreted as preliminary evidence. These alternative explanations underscore the need for future randomized controlled trials with active control groups to isolate the specific effects of MI from these confounding factors.

## 5 Conclusion

In conclusion, this pilot feasibility study suggests that telephone-delivered MI is a promising, accessible intervention for alleviating the significant psychosocial distress experienced by DR-TB patients. The non-significant finding regarding medication adherence, informed by robust objective measurement, refines our understanding of the intervention’s target population and primary benefits.

Future research should prioritize randomized controlled trials with active control groups, more diverse populations with varying baseline adherence, and longer follow-up periods to establish efficacy, confirm causal relationships, and identify the patients most likely to benefit from specific intervention components.

## 6 Limitations

The interpretability and generalizability of our findings are constrained by several limitations inherent to the exploratory pilot design. As a pilot feasibility study, the findings should be interpreted as preliminary and hypothesis-generating.

First, the absence of a concurrent control group precludes definitive causal attribution of the observed changes to the MI intervention alone, as factors such as Hawthorne effects, natural history, concurrent clinical care, or placebo effects may have contributed (as discussed in Section 4.5).

Second, the relatively small sample size, recruited from a single center, limits statistical power and generalizability. This is compounded by potential selection bias: our sample possessed a notably high education level (over 50% held a university degree or higher) and lacked chronic comorbidities, which may limit generalizability to less-educated, older, or medically comorbid DR-TB populations.

Third, the lack of a formal *a priori* power calculation and the decision not to adjust for multiple comparisons, while justified for this feasibility study, increase the risk of Type II error and necessitate cautious interpretation of the *p*-values, particularly for non-significant findings like medication adherence.

Fourth, the study was not powered to conduct formal subgroup analyses to explore whether intervention effects varied by factors such as gender, age, treatment stage, or education level. Additionally, participants were enrolled at varying time points since treatment initiation (reflecting real-world clinical diversity). While such analyses could provide valuable insights for tailoring interventions, they would be prone to Type I and II errors in this exploratory sample.

Future confirmatory studies with larger sample sizes, control groups, and multiple assessment points should prospectively examine these temporal dynamics and potential effect modifiers.

## Data Availability

The original contributions presented in the study are included in the article/Supplementary material, further inquiries can be directed to the corresponding authors.
