# Supplementary material for: Alleviating psychosocial distress in drug-resistant tuberculosis: a pre-post study on telephone-delivered motivational interviewing
Source: Front Public Health. 2026 May 8;14:1817457. doi: 10.3389/fpubh.2026.1817457 (PMC13194114; doi:10.3389/fpubh.2026.1817457)

**Informed Consent Form**

Dear Patient:

You are invited to participate in a study conducted by the Public Health Clinical Center of Chengdu. This study aims to evaluate the effectiveness of a telephone-delivered motivational interviewing intervention in alleviating loneliness, depression, and anxiety among patients with drug-resistant tuberculosis.

This informed consent form provides you with necessary information to help you decide whether or not to participate in this study. Your participation is completely voluntary. This study has been approved by the Medical Ethics Committee of the Public Health Clinical Center of Chengdu (Approval No.: PJ-K2020-56-01). If you agree to participate, please read the following instructions carefully:

**Study Purpose:**

Patients with drug-resistant tuberculosis often experience significant psychological distress, including loneliness, anxiety, and depression during treatment. These emotional challenges not only affect patients' quality of life but may also impact treatment adherence and prognosis. This study aims to implement a telephone-delivered motivational interviewing intervention to help patients alleviate psychological distress, improve mental health status, and enhance treatment confidence, thereby providing scientific evidence for integrating psychological support services into routine clinical care.

**Study Methods:**

This study employs a single-group pre-post design and plans to enroll 74 patients with drug-resistant tuberculosis. The study period is 16 weeks, during which you will receive four telephone-based motivational interviewing sessions (at Weeks 2, 4, 8, and 12), each lasting approximately 30-40 minutes. The interviews will focus on your psychological status, treatment experiences, and emotional regulation, aiming to enhance your intrinsic motivation and improve your mental well-being.

You will be asked to complete the following assessments at baseline (Week 0) and post-intervention (Week 16):

General demographic information and clinical data;

Psychological status questionnaires (including loneliness, anxiety, and depression);

Sleep quality assessment;

Medication adherence assessment (based on pill count).

The questionnaires and assessments will take approximately 30 minutes to complete and will not interfere with your regular treatment or daily life.

**Confidentiality Statement:**

Your privacy will be strictly protected throughout this study. All information collected will be used solely for research purposes and will not be disclosed in any form that identifies you personally. Study results will be statistically analyzed and published without revealing individual identities.

**Contact Information：**

If you have any questions or concerns, please contact:

Principal Investigator: Xie Fanghui

Tel: 15982001676

Email: 471987472@qq.com

For ethical concerns, contact the Ethics Committee of the Public Health Clinical Center of Chengdu at 028-64369118.

**Informed Consent Signature Page:**

I have read this informed consent form, and the researcher has explained the purpose, content, procedures, potential risks, and benefits of this study to me in detail and has answered all my questions. I understand that my participation in this study is voluntary, and I agree to participate.

Signature of participant: _________________________

Date: _________________________

Signature of researcher: _________________________

Date: _________________________

**1. Loneliness Scale (UCLA Loneliness Scale, Version 3)**

Instructions: The following statements describe how people sometimes feel. For each statement, please indicate how often you feel the way described by circling one of the responses below.

| Item | Never | Rarely | Sometimes | Always |
| --- | --- | --- | --- | --- |
| 1. How often do you feel that you are in harmony with the people around you? |  |  |  |  |
| 2. How often do you feel that you lack companionship? |  |  |  |  |
| 3. How often do you feel that there is no one you can turn to? |  |  |  |  |
| 4. How often do you feel alone? |  |  |  |  |
| 5. How often do you feel part of a group of friends? |  |  |  |  |
| 6. How often do you feel that you have a lot in common with the people around you? |  |  |  |  |
| 7. How often do you feel that you are no longer close to anyone? |  |  |  |  |
| 8. How often do you feel that your interests and ideas are not shared by those around you? |  |  |  |  |
| 9. How often do you feel outgoing and friendly? |  |  |  |  |
| 10. How often do you feel close to people? |  |  |  |  |
| 11. How often do you feel left out? |  |  |  |  |
| 12. How often do you feel that your relationships with others are not meaningful? |  |  |  |  |
| 13. How often do you feel that no one really knows you well? |  |  |  |  |
| 14. How often do you feel isolated from others? |  |  |  |  |
| 15. How often do you feel you can find companionship when you want it? |  |  |  |  |
| 16. How often do you feel that there are people who really understand you? |  |  |  |  |
| 17. How often do you feel shy? |  |  |  |  |
| 18. How often do you feel that people are around you but not with you? |  |  |  |  |
| 19. How often do you feel that there are people you can talk to? |  |  |  |  |
| 20. How often do you feel that there are people you can turn to? |  |  |  |  |

**2.** **Hamilton Anxiety Rating Scale (HAMA)**

Below is a list of phrases that describe certain feeing that people have. Rate the patients by finding the answer which best describes the extent to which he/she has these conditions, Select one of the five responses for each of the fourteen questions.

0= Not present, 1= Mild, 2= Moderate, 3= Severe, 4= Very severe.

| Symptom Cluster / Item | Description | Scoring (Circle One) |
| --- | --- | --- |
| 1. Anxious Mood | Worries, anticipation of the worst, fearful anticipation, irritability. | 0 1 2 3 4 |
| 2. Tension | Feelings of tension, fatigability, startle response, moved to tears easily, trembling, feelings of restlessness, inability to relax. | 0 1 2 3 4 |
| 3. Fears | Of dark, strangers, being left alone, animals, traffic, crowds. | 0 1 2 3 4 |
| 4. Insomnia | Difficulty in falling asleep, broken sleep, unsatisfying sleep and fatigue on waking, dreams, nightmares, night terrors. | 0 1 2 3 4 |
| 5. Intellectual (Cognitive) | Difficulty in concentration, poor memory. | 0 1 2 3 4 |
| 6. Depressed Mood | Loss of interest, lack of pleasure in hobbies, depression, early waking, diurnal swing. | 0 1 2 3 4 |
| 7. Somatic (Muscular) | Pains and aches, twitching, stiffness, myoclonic jerks, grinding of teeth, unsteady voice, increased muscular tone. | 0 1 2 3 4 |
| 8. Somatic (Sensory) | Tinnitus, blurring of vision, hot and cold flushes, feelings of weakness, pricking sensation. | 0 1 2 3 4 |
| 9. Cardiovascular Symptoms | Tachycardia, palpitations, pain in chest, throbbing of vessels, fainting feelings, missing beat. | 0 1 2 3 4 |
| 10. Respiratory Symptoms | Pressure or constriction in chest, choking feelings, sighing, dyspnea. | 0 1 2 3 4 |
| 11. Gastrointestinal Symptoms | Difficulty in swallowing, wind, abdominal pain, burning sensations, abdominal fullness, nausea, vomiting, borborygmi, looseness of bowels, loss of weight, constipation. | 0 1 2 3 4 |
| 12. Genitourinary Symptoms | Frequency of micturition, urgency of micturition, amenorrhea, menorrhagia, development of frigidity, premature ejaculation, loss of libido, impotence. | 0 1 2 3 4 |
| 13. Autonomic Symptoms | Dry mouth, flushing, pallor, tendency to sweat, giddiness, tension headache, raising of hair. | 0 1 2 3 4 |
| 14. Behavior at Interview | (General) Fidgeting, restlessness or pacing, tremor of hands, furrowed brow, strained face, sighing or rapid respiration, facial pallor, swallowing, belching, brisk tendon reflexes, dilated pupils, exophthalmos. | 0 1 2 3 4 |
| Scoring Guide: 0=Not present; 1=Mild; 2=Moderate; 3=Severe; 4=Very severe/Disabling. | | Total Score: |

**3. Self-Rating Depression Scale (SDS)**

Instructions: Please read each statement and decide how much of the time the statement describes how you have been feeling during the past several days.

□ A: A little of the time (Occasionally)

□ B: Some of the time (Sometimes)

□ C: Good part of the time (Often)

□ D: Most of the time (Continuously)

| Item | | A | B | C | D |
| --- | --- | --- | --- | --- | --- |
| 1. I feel down-hearted and blue. | |  |  |  |  |
| 1. Morning is when I feel the best. | |  |  |  |  |
| 1. I have crying spells or feel like it. | |  |  |  |  |
| 1. I have trouble sleeping at night. | |  |  |  |  |
| 1. I eat as much as I used to. | |  |  |  |  |
| 1. I enjoy looking at, talking to and being with attractive women/men. | |  |  |  |  |
| 1. I notice that I am losing weight. | |  |  |  |  |
| 1. I have trouble with constipation. | |  |  |  |  |
| 1. My heart beats faster than usual. | |  |  |  |  |
| 1. I get tired for no reason. | |  |  |  |  |
| 1. My mind is as clear as it used to be. | |  |  |  |  |
| 1. I find it easy to do the things I used to. | |  |  |  |  |
| 1. I am restless and can't keep still. | |  |  |  |  |
| 1. I feel hopeful about the future. | |  |  |  |  |
| 1. I am more irritable than usual. | |  |  |  |  |
| 1. I find it easy to make decisions. | |  |  |  |  |
| 1. I feel that I am useful and needed. | |  |  |  |  |
| 1. My life is pretty full. | |  |  |  |  |
| 1. I feel that others would be better off if I were dead. | |  |  |  |  |
| 1. I still enjoy the things I used to do. | |  |  |  |  |
|  |  |  |  |  |  |

**4. Pittsburgh Sleep Quality Index (PSQI)**


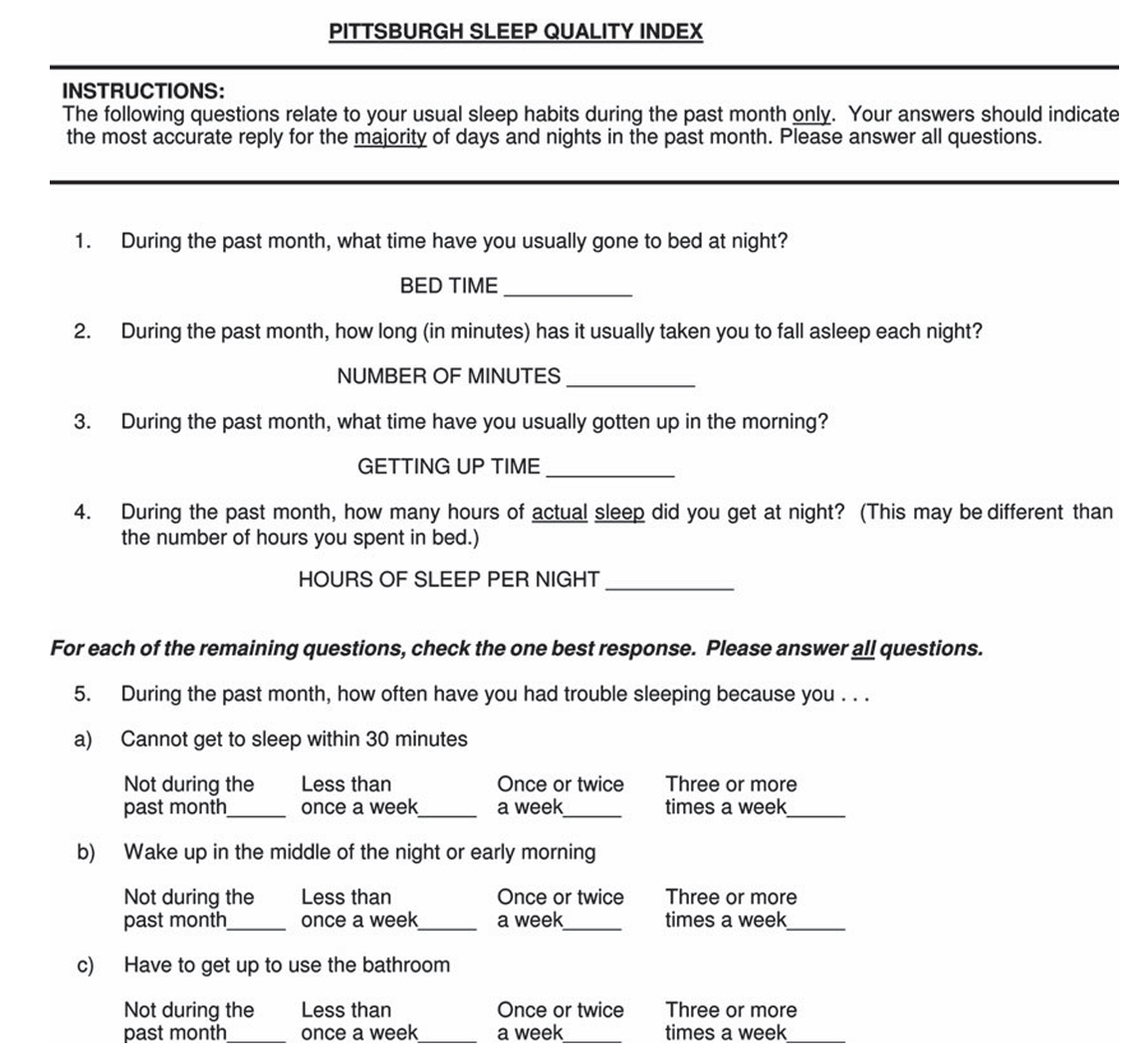

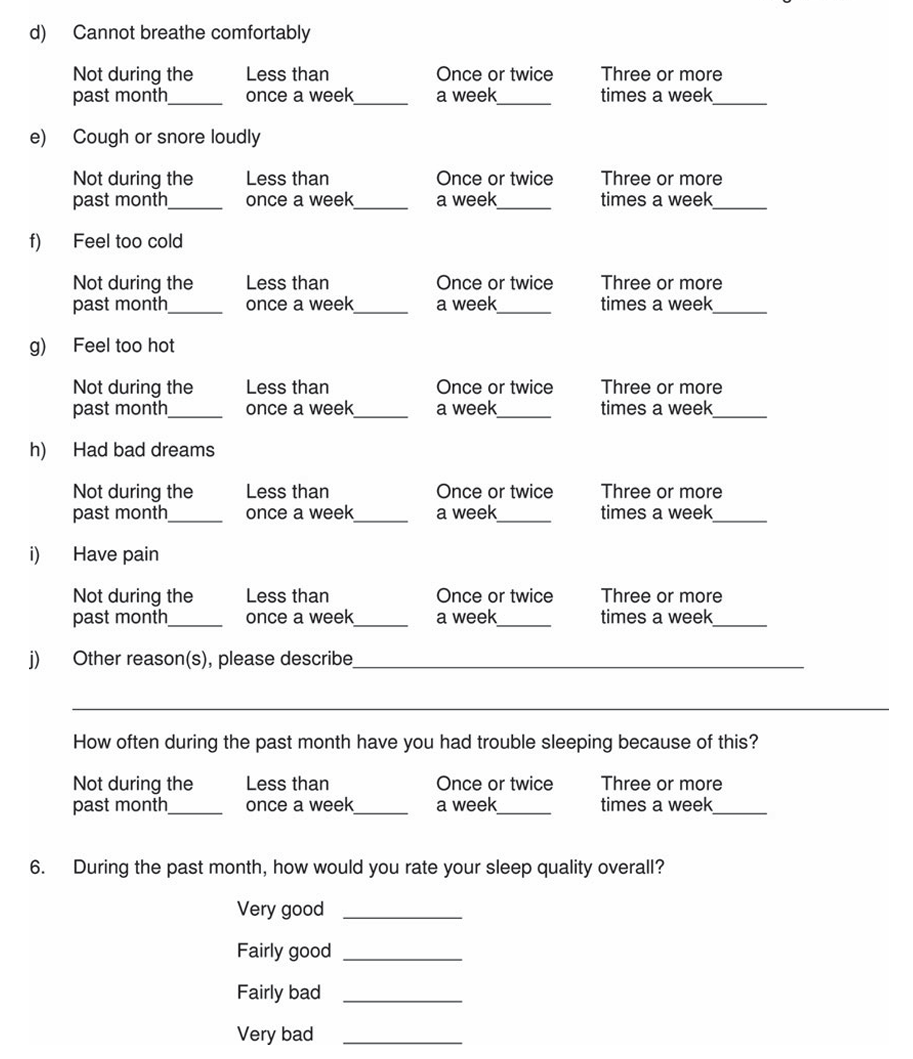


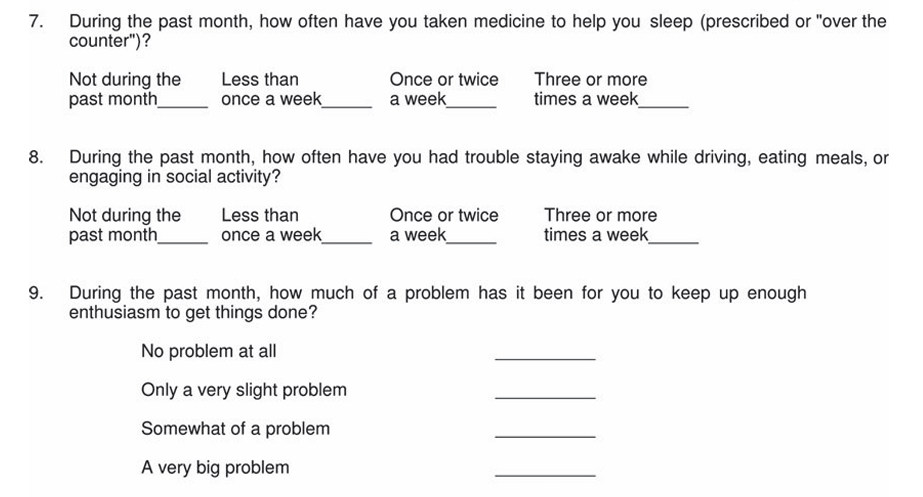

Supplement: Supplementary file 4 [file Table_2.docx]
